# Accuracy of Methylene Blue Test as Single Technique for Sentinel Lymph Node Biopsy in Early Stages Breast Cancer

**DOI:** 10.31557/APJCP.2021.22.9.2765

**Published:** 2021-09

**Authors:** Dedy Hermansyah, Yolanda Rahayu, Arjumardi Azrah, Gracia Pricilia, Desiree Paramita, Edwin Saleh Siregar, Sufida Sufida, Albiner Simarmata

**Affiliations:** 1 *Oncology Surgery Division of Department of Surgery, Faculty of Medicine, Universitas Sumatera, Indonesia. *; 2 *Surgery Division of Department of Surgery, Faculty of Medicine, Universitas Sumatera Utara, Indonesia. *; 3 *Department of Anatomical Pathology, Faculty of Medicine, Universitas Sumatera Utara, Indonesia. *

**Keywords:** Methylene blue, lymph node biopsy, breast cancer

## Abstract

**Aim:**

Sentinel Lymph Node Biopsy (SLNB) establishes as a gold standard for diagnostic lymph node involvement in early breast cancer. Most of the developed country does not have radiotracer and nuclear medicine facilities. Unless in Indonesia there is Methylene Blue as an alternative agent for SLNB. This study measure accuracy of sentinel lymph node biopsy as a single technique using the Methylene Blue test.

**Methods:**

This cross-sectional study enrolled 60 female patients with breast cancer stage I-II. We performed SNB using 2-5 cc of 1% Methylene-blue dye (MBD) injected to periareolar tissue and proceeded with axillary lymph nodes dissection (ALND). The histopathology results of sentinel nodes (SNs) and axillary lymph nodes (ALNs) analyze for diagnostic value assessments.

**Results:**

The identification rate of SN was 97.62 %, and the median number of identified SNs was 4 (2-7). Sentinel node metastasis was found in (19/60) % cases and % of them were macrometastases. The sensitivity and specificity of MBD were 91.67% and 96.67% respectively. The negative predictive value (NPV) of SNs to predict axillary metastasis was 96.67% (95% CI, 81-99%).

**Conclusion:**

Injection of 1% MBD as a single technique in breast cancer SNB has a favorable identification rate and predictive value.

## Introduction

One of the most accurate prognostic factors in breast cancer is axillary lymph node metastasis. Sentinel lymph node biopsy is a minimally invasive technique to confirm regional lymph node metastasis in cancer patients with less morbidity such as lymphedema, axillary web syndrome, chronic pain, and loss of sensation (Nieweget al., 2015). The SNB is well known as the gold standard diagnosis for axillary lymph node metastasis inpatient without axillary lymph node metastasis with clinical or radiology tests. Since Krag introduced SNB for breast cancer in 1993, we have found many innovations for this technique (Krag, et al., 1993) Over the past 27 years, sentinel node biopsy in breast cancer patients has become an exciting research topic. Many studies have shown that SLNB accurately predicts axillary lymph node status and associate with less morbidity than ALND completion (Zahoor et at., 2017). Results from international breast cancer centers show that, with the use of optimal techniques, SLNB predicts axillary nodal status with high accuracy and low clinical false-negative rates (Lyman, et al, 2014). There is new technique such as Indocyanine green (IC-green), Super-paramagnetic Iron Oxide (SPIO), and CEUS with micro-bubbles as an alternative to the most common technique with a dual tracer of patent blue dye and isotope (Tc99).

In a developing country like Indonesia, Radiocolloid or IC-green is not yet available; therefore, MBD alone is used to establish the diagnosis. We do sentinel lymph node biopsy using lymphatic mapping techniques such as blue-dye, radiotracer, or a combination of both. In developing countries, we carry sentinel lymph node biopsy out using blue dye only. Limited access to patent blue dye and radioisotope tracer is the major problem in conducting SLNB in Indonesia. One of the most accurate prognostic factors in breast cancer is axillary lymph node metastasis. The gold standard diagnosis for axillary lymph node metastasis is Indocyanine green (IC-green) and Methylene-blue dye (MBD) injection. 

Limited access to PBD and radioisotope tracer is the major problem to perform SNB in Indonesia. Unfortunately, many hospitals in Indonesia do not currently have the ability or qualifications to provide nuclear medicine and equipment. Not to mention our geographic distribution of the population, the availability and cost to provide nuclear medicines, or gamma probes in every hospital have contributed to the difficulty of administering SNB. Recently, we have used 1% MBD alone for us to conduct a study to overcome the limitation to perform SNB. Although the radiation exposure during SNB using radioisotopes still limited and is safe for pregnant surgeons and patients. Concern about the hazards of radiation exposure is also an obstacle for the use of the combined method. In this study, we aimed to evaluate our results of sentinel lymph node biopsy (SLNB) with methylene blue in patients with early-stage breast cancer.

The study conducted in our institution had an aim to determine the procedure validity of SLN with methylene blue alone. Considering our country, it gives the opportunity of performing SLNB even in regional away from the country-city center.

## Materials and Methods

This is cross-sectional research that was conducted in Universitas Sumatera Utara Teaching Hospital. The sample was early breast cancer patients that underwent sentinel lymph node biopsy. Then, the tissue was evaluated to find lymph node metastasis. Sixty patients fit into the inclusion criteria participated in the research. Both mastectomy a nd breast conservation surgery patients were equally eligible with SLNB. All patients undergoing the study gave their informed consent for MBD 1% 2.5 cc periareolar or combination with 2cc injection peritumoral for tumor with size >3cm and superolateral position injection of MBD 1% and the surgical procedure to be performed. The institutional review board approved the trial. 

A pathologist carefully identified and evaluated all blue SLN specimens. All lymph nodes in the specimen identify and dissected from the surrounding tissue by the pathologist. We accessed the number of nodes and their dimensions. SLNs were stained with hematoxylin and eosin (H&E). SLNBs were sectioned and examined for tumor cells.

## Results

**Figure 1:**
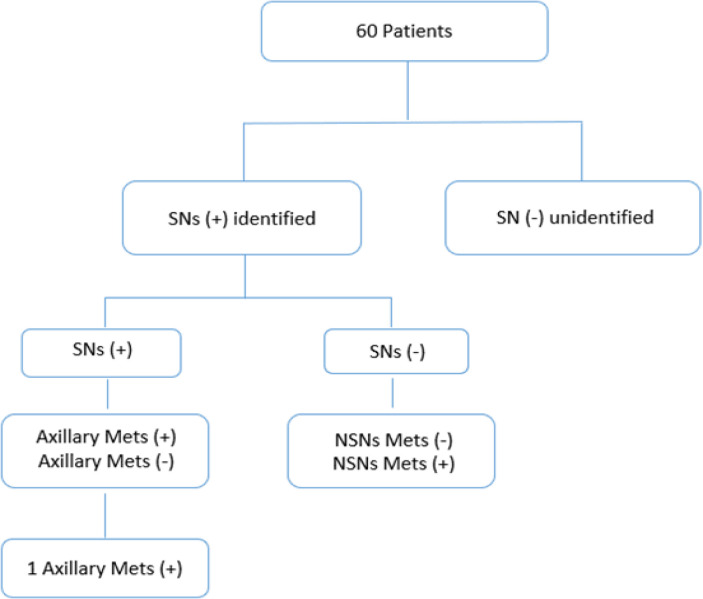
Patient Flowcart for Recruitment and SN Assessment to Predict Axillary Metastasis

From 60 samples, the identified sentinel nodes divided into positive and negative sentinel nodes, then recruited the axillary metastasis and NSN metastasis (Figure 1). From Table 1, there were 10 (16,67%) grade I patients, 13 (21,67%) grade II patients and 37 (61,67%) grade III patients. Based on its histopathology result, there were 59 (98,33%) patients with non-specific type breast cancer and 1 (1,67%) patient with invasive lobular carcinoma. There were more T2 patients (51 patients; 85,00%) compare to T1 (9 patients;15,00 %) and N1 and N2 were 60 patients (100%) and 0 patients (0%) respectively. From Table 2, six patients had macro-metastasis while 13 patients had micro-metastasis. In the positive sentinel lymph node, there were 7 patients have 1-2 lymph nodes, while the other 12 patients have over 2 sentinel nodes. From Table 3, we found most of negative sentinel nodes patients showed no metastasis from histopathology results. In Table 4, we found sensitivity in this research is 94.74%, only 1 sample is a false negative while specificity is 97.56%. PPV and NPV in the diagnostic test also yield the same result, which is 94.74% and 95.56% respectively. The false-negative of our research is 1.6% . The confidence interval in this research is 95%. We found no allergic reaction, skin necrosis, or inflammation after the procedure in whole samples.

**Table 1:** Patients’ Characteristics

Variable	
Total patients	60
Mean age ±SD	49.5 ±8.01
Lymphovascular Involvement (%)	
Yes	22 (36,67%)
No	38 (63,33 %)
Histopathology Grading	
Grade I	10 (16,67%)
Grade II	13 (21,67%)
Grade III	37 (61,67%)
TNM	
T1	9 (15,00%)
T2	51 (85,00%)
N1	60 (100%)
N2	0 (%)
M0	60 (100%)
M1	0 (0%)
Histopathology	
Invasive Lobular	1 (1,67%)
NOS/IDC/NST	59 (98,33%)
IHC	
ER/PR	41 (68,33%)
HER-2	12 (20,00%)
TNBC	7 (11,67%)

**Table 2:** Sentinel Lymph Node Characteristics in Patients with Metastasis

SN Characteristic		Number	Percentage
Positive SN count	1-2	7	36,84%
	> 2	12	63,16%
Metastasis type	Macrometastases	6	31,58%
	Micrometastases	13	68,42%

**Table 3:** Sample Diagnostic Test

Sentinel Node	Histopathology		Total
	(+)	(-)	
(+)	18	1	19
(-)	1	40	41
	19	41	60

**Table 4:** Sentinel Nodes Diagnostic Test

Specificity	Sensitivity	PPV	NPV
97.56	94.74	94.74	95.56
(82.8%-99.9%)	(61.5%-99.8%)	(61.4%-98.7%)	(81.6%-99.5%)

## Discussion

The sentinel lymph node (SLN) is the first lymph node to drainage the entire lymphatics of the breast. Since metastatic breast cancer cells travel via this route, an SLN free of metastatic cancer predicts the status of the remaining axillary nodes has also negative for metastasis. The SLNB serves as a stand-alone method for determining axillary nodal status, providing physicians with the ability to distinguish positive axillary lymph nodes in a relatively simple, safe, rational, and accurate fashion.

Our patients’ characteristics have a mean age of 49.5±8.01 years old, other studies from Turkey and Indonesia have the same mean age (50 years old) early breast cancer patients, but Varghese et al. have a different mean age of early breast cancer patients (58.5 years old). The clinicopathological of our patients with 63.33% lymphovascular involvement, but another research in China has no involvement of lymphovascular invasion (92.2%). For most of our patients with histopathological grade III (61.67%), Paulinelli from Brasil has a different histopathological grade of the patients with > 50% patients is grade II (Paulinelli et al., 2017). Ushadevi et al., (2017) also has 52.8% histopathological grade II patients. Most of our patients have tumor size >20mm (T2) 85%, but most of Brahma and Varghese patients have tumor size <20 mm (T1) all the patients are N1 and there were no patients with metastasis. The histopathology of our patients is invasive lobular (1.67%) and NOS/Invasive Ductal Carcinoma (98.33%). From another research, 92.2% of patients of Paulinelli et al., (2017) have histopathology invasive ductal carcinoma. Most of our patients are ER/PR subtype (68.33%). Li Huang et al. also has most of the patients with ER/PR subtype (13.6% Luminal A and 61.3% Luminal B patients). 

Our research found 7 patients with 1-2 positive SN and 12 patients with over 2 positive SN. We also found 6 patients with macro-metastasis and 13 patients with micro-metastasis. It means that 48 of our patients didn’t need the ALND procedure and could have saved a lot of patients from having the risk of lymphedema and other morbidities post ALND. Mathelin et al., (2009) found 11 micro-metastasis and 29 macro-metastases from 100 total early breast cancer patients. Brahma et al., found 43 patients with 1-2 positive SN and 4 patients with more than 2 positive SN, also found 42 patients with macro-metastasis and 5 patients with micro-metastasis from the total of 96 early breast cancer patients. In this study, we could not find SNs in 8 patients. Only 1 patient in our research with negative SN but positive histological examination, we still found factors that related to this condition, some literature found that age, body mass index (BMI), tumor size, location, grade, type of the previous biopsy, SNB technique, and surgeon’s experience influence SN identification. The increased fatty tissue in the breast among older patients may decrease lymphatic flow and failures to identify SNs.

Research by Golshan et al., (2006) stated that methylene blue has high sensitivity and specificity in detecting sentinel lymph node metastasis, which is like this research where sensitivity is 91.6% and specificity is 96.6%. Nour (2004) in his research, also stated that methylene blue alone can identify sentinel lymph node metastasis in breast cancer patients. Mathelin et al. in 2009 said that methylene blue has very good accuracy.

Brahma et al., in 2017 conducted similar research in 96 patients with a sensitivity of 92% and specificity of 100%. PPV and NPV of the research are 100% and 90% respectively. Li et al., (2017) did other research that supported the same idea in 2014 with sensitivity 87%, NPV 91%, and AR 94%. Another research by Ozdemir et al., (2014) in Turkey has a sensitivity of 85% and specificity of 100% also PPV and NPV are 90% and 100% respectively. 

Gupta et al., (2020) from India in 2019 researched 2 groups of early stages breast cancer patients, group 1 is the patient with only blue dye only group, and group 2 was the combination of methylene blue and radiocolloid. They found the sensitivity, specificity, and NPV of group 1 are 75%, 95,45%, and 91,3%. In the group 2 they found sensitivity, specificity, and NPV are 83,33%, 91,67%, and 95,65%. 

A study by Ge et al., (2011) compare methylene blue and carbonic nanoparticles in detecting sentinel lymph node biopsy. Carbonic nanoparticle shows better result in where its sensitivity is 98.5% and sensitivity of methylene blue was only 88.2%. Another research by Qin et al., (2019) comparing methylene blue dye combine with indocyanine green and carbonic nanoparticle showed that a combination of methylene blue and indocyanine green has 100% of detection rate and carbonic particle only 96.7% of detection rate.

Li et al., (2018) research compared the combined IR and FNR of SNB according to different MBD doses. The combined IR for the studies that used a 2-ml injection of MBD was 90% for the studies that used a 5-ml injection of MBD, the combined IR was 92%. We detected no significant difference between the two groups of studies (P = 0.980). The combined FNR for the studies that used a 2-ml injection of MBD was 11%, for the studies that used a 5-ml injection of MBD, the FNR was 10%. We detected no significant difference in the FNR of SNB between the studies that used 2 ml MBD and those that used 5 ml (P = 0.555).

In this study, we didn’t find side effects from MB1% injection to all of our patients. This research shows that methylene blue is safe to use, as there is a 0 to none anaphylactic reaction in methylene blue application. We have never reported the anaphylactic reaction in methylene blue dye injection for sentinel lymph node identification in breast cancer. Compare to lymphazurin, there has been a reported anaphylactic reaction of about 1-3%. Comparing to Mathelin’s study with intraparenchymal subareolar injection, MBD was safe, with no major adverse events being noticed and no axillary recurrences being diagnosed after a mean follow-up of 28 months. In particular, we observed no allergic or anaphylactic reactions during the SLN procedure. Teknos et al., (2008) reporting pulmonary edema during an SLN procedure using MBD. In the present clinical trial, they observed no necrotic skin lesions, in contrast to published data. Stradling et al. reported 5 necrotic skin lesions (21%) after the injection of 3 to 5 mL of MBD (full strength 1%, 10 mg/mL) in a series of 24 patients with MBD were injected into both the parenchyma and the skin. None of the patients required surgical debridement. Salhab et al., (2011) reported severe skin and fat necrosis. with a peritumoral MBD injection, but the concentration of the MBD does not mention in the case report. In the recent prospective study of Saha et al., (2015) comparing IBD and MBD, 7% of skin necrosis observe after intraparenchymal and intradermal area injection of 3-5 mL of 1% MBD. Zakaria et al., (2007)analyzed 381 patients receiving MBD and described 5 cases of skin necroses. One of these patients developed full-thickness skin necrosis that required excision. In the remaining 4 cases, the areas of skin necrosis healed without intervention. The absence of skin necrosis in the present study could probably be explained by the use of deep intraparenchymal injections with a lower volume of MBD (2 mL of MBD, 10 mg/mL). In the present series, the only minor adverse effect was temporary tattooing of the breast which was noticed in 12 cases (11%), with complete resolution in each case.

Although many centers have moved on to the combination technique, we should consider using blue dye alone whenever a radioactive tracer is not available instead of accepting the high morbidity of complete axillary dissection. Cost issues, especially in countries where most of the population does not have insurance cover, should not limit the use of SLNB. It is essential that the surgeon adapts to the facilities available and be ontologically sound. However, concerns regarding the availability of nuclear medicine facilities have dampened its widespread use in developing countries. With this study and literature review, we have tried to address this debatable topic, and we found that 1% MDB has a favorable to diagnose sentinel lymph node metastases.

The learning process and learning curve are important things to the success rate of SLNB. During the learning process of sentinel lymph node biopsy, we should perform axillary dissection after identification of the sentinel lymph node. We should compare the results of sentinel node biopsy with axillary dissection results. While at least 90% sentinel lymph node detection rate and less than 5% false-negative rate shows that only sentinel lymph node biopsy without axillary dissection can be made. Tafra et al., (2008) reported that 30 cases are adequate for the learning phase. In the ALMANAC study, they have reported that at least 40 patients are required. In the Ozdemir et al., (2014) study of 30 cases, the sentinel lymph node detection rate was over 90% even though the false-negative rate was above 5%. We believe that as the number of patients increases, the false negativity rate will decrease to the desired level. These rates prove we need to continue with axillary dissection after sentinel lymph node biopsy, and that we could not reach ideal results yet.

In conclusion, injection of 1% MBD as a single technique in breast cancer SNB has a favorable identification rate and predictive value which were 94.74% and 94.74% respectively.

## Author Contribution Statement

The authors confirm contribution to the paper as follows: study conception and design: Dedy, H and Albiner, S; data collection: Arjumardi, A., Yolanda, R., and Sufida; analysis and interpretation of results: Dedy, H., Gracia, P., and Edwin, S; draft manuscript preparation: Dedy, H., and Desiree, P. All authors reviewed the results and approved the final version of the manuscript.

## Conflicts of Interest

None.
